# The Design of a Piecewise-Integrated Composite Bumper Beam with Machine-Learning Algorithms

**DOI:** 10.3390/ma17030602

**Published:** 2024-01-26

**Authors:** Seokwoo Ham, Seungmin Ji, Seong Sik Cheon

**Affiliations:** 1 Innowill Co., Ltd., Daejeon 34325, Republic of Korea; wooya@smail.kongju.ac.kr; 2 Department of Mechanical Engineering, Kongju National University, Cheonan 31080, Republic of Korea; sm.ji@smail.kongju.ac.kr

**Keywords:** composite material, bumper beam, machine learning, stacking sequence, piecewise-integrated composite

## Abstract

In the present study, a piecewise-integrated composite bumper beam for passenger cars is proposed, and the design innovation process for a composite bumper beam regarding a bumper test protocol suggested by the Insurance Institute for Highway Safety is carried out with the help of machine learning models. Several elements in the bumper FE model have been assigned to be references in order to collect training data, which allow the machine learning model to study the method of predicting loading types for each finite element. Two-dimensional and three-dimensional implementations are provided by machine learning models, which determine the stacking sequences of each finite element in the piecewise-integrated composite bumper beam. It was found that the piecewise-integrated composite bumper beam, which is designed by a machine learning model, is more effective for reducing the possibility of structural failure as well as increasing bending strength compared to the conventional composite bumper beam. Moreover, the three-dimensional implementation produces better results compared with results from the two-dimensional implementation since it is preferable to choose loading-type information, which is achieved from surroundings when the target elements are located either at corners or junctions of planes, instead of using information that comes from the identical plane of target elements.

## 1. Introduction

The improvement in the crashworthiness of automobiles cannot be overestimated. The National Highway Traffic Safety Administration estimated that over 60% of cases among 11,927 fatal passenger car accidents were due to frontal collisions, and frontal collisions were found to be the most harmful accident in 2021 [1]. This, together with a range of environmental concerns and social pressures backed by legislation, has led and will continue to lead to highly innovative design involving lighter materials such as light metals and composites [2,3,4,5]. The use of composite materials, however, is also governed by their ability to meet the increasing demands for crashworthiness, with the ultimate goal being the reduction of occupant harm and/or vehicle damage [5,6]. During the frontal collision, a bumper beam is the first exposed part in the passenger car onto the barrier or the other car; therefore, it is crucial to design the bumper beam in order to maximise its load-resisting capability [6,7,8]. In the meantime, it has been found that composite products, which are designed based on conventional methods with a simple combination of several stacking sequences, are no longer competitive. The best way to achieve structural performances beyond the limits without a weight-saving effect is by applying either artificial intelligence techniques or machine learning models [9].

Of particular interest to this study is designing a composite bumper beam with the help of machine learning models in order to improve its load-resisting capability beyond the current limit. The objective is to improve the crashworthiness of the composite bumper beam, since crashworthiness depends on external load-resisting capability at a certain level of deformation.

Kim et al. [10] suggested an optimised design for a hybrid glass/carbon mat thermoplastic composite bumper beam and showed effective weight reduction with improved impact performance. Belingardi et al. [11] suggested lightweight materials as well as a manufacturing process for a composite bumper beam. Liu et al. [12] applied particle swarm optimisation to the design of an automotive composite bumper system. Rao et al. [13] analysed the crash behaviour of bumper beams made of several materials with different thicknesses, and the material with the highest yield strength was recommended for manufacturing bumper beams. Kong et al. [14] performed a structural design on an automotive hood with a natural flax fibre/vinylester composite. Wang et al. [15] proposed a bumper beam with periodic inner hexagonal cellular structures in order to achieve negative Poisson’s ratio characteristics for improving energy absorption capability during crashes. As shown so far, there have been many studies on improving the characteristics by changing the material of the bumper beam; however, the entire range of composite bumper beams has been made based on a conventional single-style stacking sequence without applying other types of stacking sequences over the entire region of the composite structure.

Jeong et al. [16] proposed a novel concept of PIC (piecewise-integrated composite), in which the loading type of bumper beam was analysed and dissimilar stacking sequences, i.e., tension-dominant, compression-dominant, and shear-dominant stacking sequences, were assigned into five macro sub-regions of the composite bumper beam. Even though a PIC bumper beam showed a slight possibility to improve load-resisting capability, its result was far from satisfactory since external loading types altered based on a finite element size scale, i.e., each local element of the bumper beam model was experiencing different types of loading during a crash.

Consequently, it is necessary to automatically assign robust stacking sequences against different loading types to each finite element of a composite bumper beam model, and the most efficient way to assign dissimilar stacking sequences to all elements is using machine learning models.

The aim of the current work is to design a PIC bumper beam using machine learning models in order to improve its external load-resisting capability, i.e., bending strength beyond the current limit.

A preliminary FE (finite element) crash analysis, which regards the bumper test protocol suggested by IIHS (Insurance Institute for Highway Safety) [17], reveals external loading types acting on selected finite elements or reference elements of a bumper beam. An FE model and machine learning algorithms are applied in order to effectively classify the loading types of every finite element of a bumper beam. Meanwhile, training data, which allow the machine learning models to study the method of predicting loading types for every finite element, are obtained from preliminary FE crash analysis regarding the bumper test protocol by IIHS. Also, the training data in the current study should contain information about stress triaxiality along with location in the form of coordinate values. Five different types of algorithms, which underlie the machine learning model, are applied to guarantee the highest performance in view of improving the bending strength and structural lightweight effect of the product [18,19,20]. The results of the highest performance predict the loading type of each finite element, and robust stacking sequences against loading type are mapped into the entire region of bumper beam finite elements. Finally, the load-resisting ability and structural weight-saving effect of the designed PIC bumper beam are verified through FE crash analysis regarding the identical protocol, i.e., the bumper test protocol by IIHS.

## 2. Outline of the PIC Bumper Beam Design Process

The PIC is assigning different stacking sequences for each shell element with a size of 4 mm × 4 mm as a similar form of mosaic, and elements are assumed to have been perfectly bonded to each other in order to increase robustness towards various external loading types. Firstly, a preliminary FE crash analysis regarding the bumper test protocol by IIHS is carried out to obtain data, which are the input to machine learning models. For the material of the bumper beam during the preliminary FE crash analysis, aluminium alloy 7021 [21] is used since stress distribution within the beam is identical regardless of the material of the beam. Several elements among finite elements are chosen to be reference elements in order to collect stress triaxiality for judging the state of loading type, i.e., tension, compression, or shear with proper location. These data, resulting from reference elements, i.e., loading type and location, are defined as training data, which are the input to machine learning model, as explained previously.

**Figure 1:**
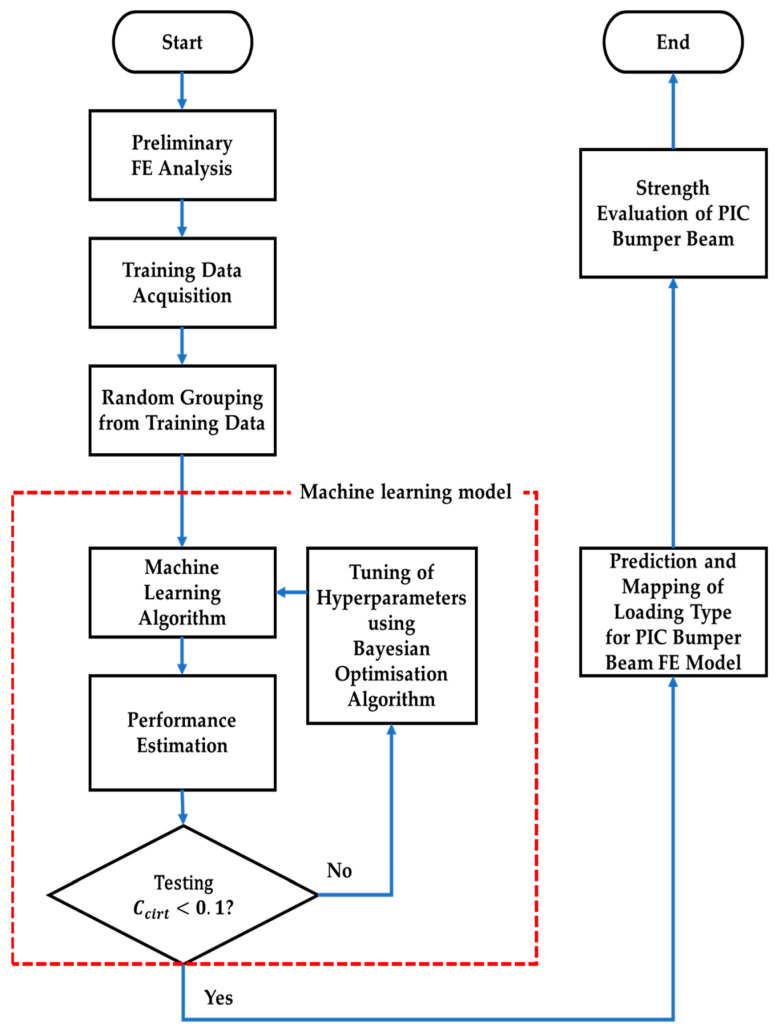
Flowchart of designing PIC bumper beams.

The acquired training data are randomly assigned to five groups, i.e., each group contains 20% of training data for generalisation’s sake [22]. One of the groups is arbitrarily chosen for testing; the others are for training, which are put into machine learning models; therefore, five trials are to be carried out per iteration, which are known to be k-fold cross-validation [23]. In the meantime, machine learning models produce different performances according to their hyperparameter values. Mean value of accuracy and ROC (receiver operating characteristic)-AUC (area under the curve) result from performance testing of machine learning algorithms. On the condition that the results are not satisfactory, hyperparameters are tuned through the Bayesian optimisation algorithm and iterations are conducted until the lowest error or the highest performance is obtained. The iterative process, i.e., training group, machine learning algorithms, performance testing, and hyperparameter tuning, are known as the machine learning model. When the results are acceptable, then loading types of unreferenced elements are able to be assigned and mapped into finite elements of the PIC bumper beam. Figure 1 shows the flowchart of the entire process for designing PIC bumper beams.

## 3. Preliminary Bumper FE Crash Analysis

FE analysis was conducted with ANSYS LS-DYNA (ANSYS, Inc., Canonsburg, PA, USA), one of the representative explicit finite element programs.

The Belytschko–Lin–Tsay quadrilateral shell element based on three integration points through the thickness direction with a 6 mm average element size [24] was selected in order to model the thin-walled structures of bumper beams, excluding hourglass mode, since it is known to be suitable for expressing the nonlinear anisotropic behaviour of thin shell structures, including complex loads and large deformations, effectively saving computation time [25].

**Figure 2:**
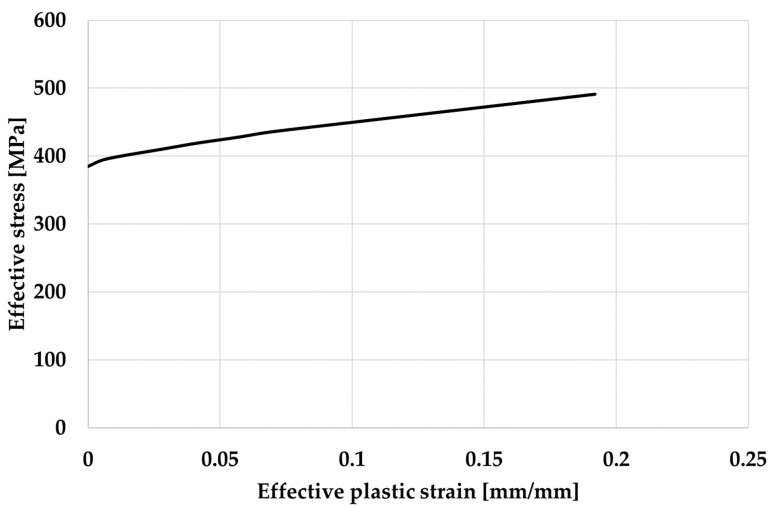
Effective stress vs effective plastic strain curve for Aluminium alloy 7021.

A material model that needs to represent an elasto-plastic behaviour with an arbitrary stress as a function of strain curve and arbitrary strain rate dependency was selected [26]. As previously noted, aluminium alloy 7021 was used for the material of the bumper beam during the preliminary FE crash analysis. The mechanical properties of aluminium alloy 7021 are summarised in Table 1 and Figure 2 [21].

**Figure 3:**
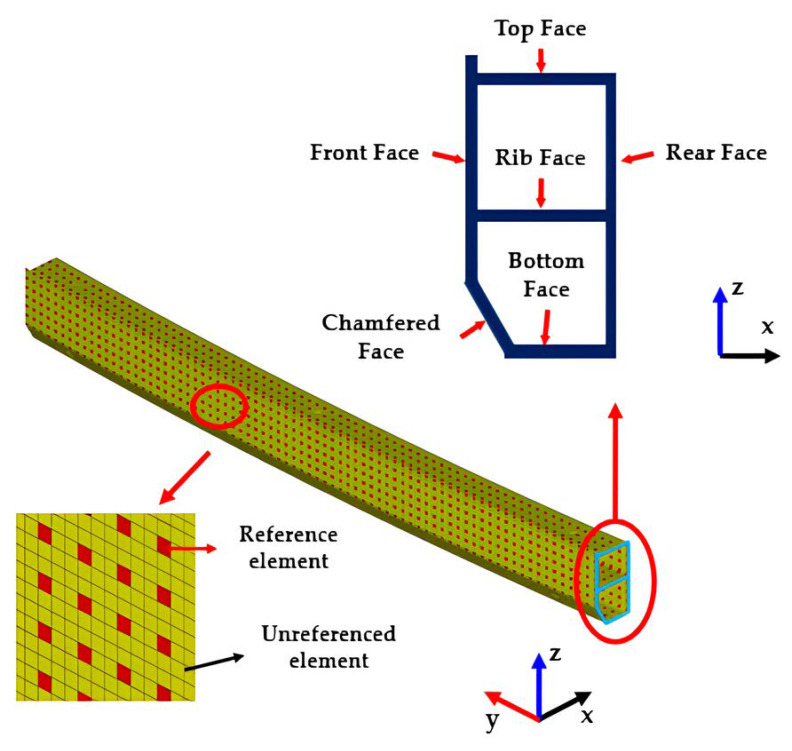
FE bumper beam model.

About 13% of the total finite elements were assigned as references in order to collect stress triaxiality for judging the state of the loading type. Stress triaxiality and coordinate location values for each reference element were included in the training data. The total number of elements in the bumper beam was about 9500, and that of the reference elements was 1210. Figure 3 shows the finite element bumper beam model, indicating reference elements and the cross-section of the bumper beam. The cross-sectional shape, total length, and radius of curvature for the bumper beam were designed with regard to the current product, which was installed in a representative average passenger car of “A” company. Actual vehicle weight was modelled as a concentrated mass element [16].

**Figure 4:**
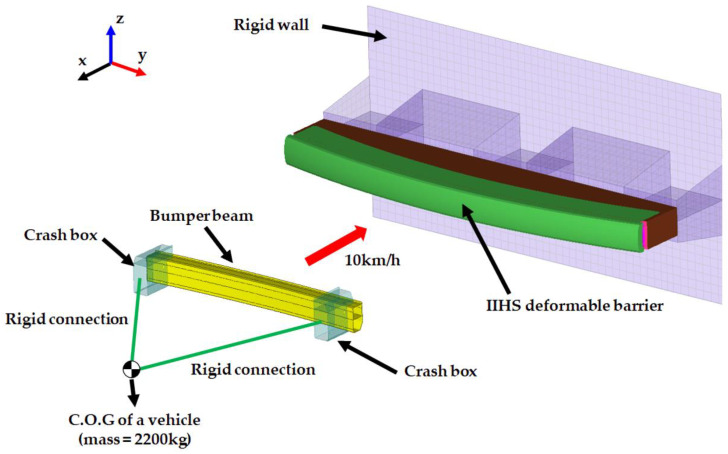
The bumper beam model and the deformable barrier for IIHS bumper crash analysis.

The IIHS bumper test was representatively used to measure the damage regions of a vehicle during a low-speed crash. It requires a vehicle as well as a deformable barrier. For the simulation, the deformable barrier was modelled with 39,800 elements based on the bumper test protocol by IIHS [17]. The FE model for an automotive bumper beam with bumper crash boxes and an IIHS standard deformable barrier is illustrated in Figure 4. Aluminium alloy 7021 was also used for the material of the bumper crash box, which is firmly connected to a rectangular cross-sectional to both ends of the bumper beam. As indicated in Figure 4, the initial speed is set to be 10 km/h, and the vehicle mass of 2200 kg is given to the specific point in which the real C.O.G. (centre of gravity) of a passenger car is located. A detailed description of the boundary condition and vehicle mass in the IIHS crash analysis is summarised in Table 2.

After finishing the analysis, it was found that there were 505 reference elements in tension, 593 in compression, and 112 in shear among 1210 reference elements, which were necessary information for preparing the training data.

## 4. Training Data Acquisition and Random Grouping

As previously mentioned, the training data, which consist of stress triaxialities and coordinate location values, are obtained from preliminary IIHS bumper beam FE analysis.

There are two different implementation methods for random grouping, i.e., two-dimensional (2-D) implementation and three-dimensional (3-D) implementation. When the 2-D implementation is used, the training data is obtained from each face of the bumper, i.e., the training data for the top, front, chamfered, bottom, rear, and rib faces, respectively. Each training data is regulated on each face, i.e., training data that is obtained from the top face is used only for predicting the loading type of reference elements located in the top face, etc. Meanwhile, training data for any face can be used for any face in 3-D implementation.

The loading type of reference elements is determined by the stress triaxiality of each element. The definition of stress triaxiality, denoted by η, is shown in Equation (1).

(1) $$\eta =\frac{\sigma_{m}}{\bar{\sigma }}$$

where $\sigma_{m}$ and $\bar{\sigma }$ are the mean principal stress and the von Mises stress, respectively [27,28]. Equations (2) and (3) show the definitions of mean principal stress and von Mises stress, respectively.

(2) $$\sigma_{m}=\frac{\left(\sigma_{x}+\sigma_{y}+\sigma_{z}\;\right)}{3}$$

(3) $$\bar{\sigma }=\sqrt{\frac{1}{2}\left[{\left(\sigma_{x}-\sigma_{y}\right)}^{2}+{\left(\sigma_{y}-\sigma_{z}\right)}^{2}+{\left(\sigma_{z}-\sigma_{x}\right)}^{2}+3\left(\sigma_{xy}^{2}+\sigma_{yz}^{2}+\sigma_{zx}^{2}\right)\right]}$$

The loading type can be classified by the stress triaxiality value. Bai and Wierzbicki [27,28] found that the dominant loading type for Al alloy 7021 is considered to be tension when η is bigger than 0.1. Also, compression is prevalent if η is smaller than −0.1. In case the η value of a specific part is bigger than −0.1 and smaller than 0.1, that part is subjected to shear loading.

**Figure 5:**
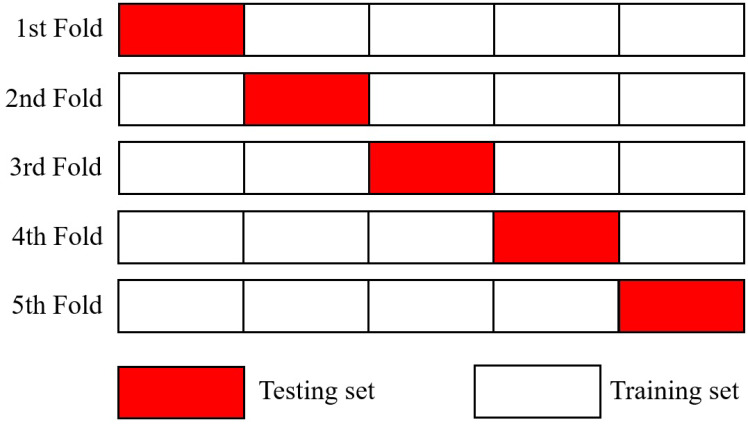
k-fold cross validation.

Acquired training data are randomly divided into five groups, i.e., each group contains 20% of training data for the sake of generalisation. As previously noticed, one of the groups is arbitrarily chosen for testing, the others for training, which are put into machine learning algorithms; therefore, five trials are to be carried out per iteration, which are known to be k-fold cross-validation, as illustrated in Figure 5.

## 5. Machine Learning Models

### 5.1. Machine Learning Algorithms

Machine learning model consists of machine learning algorithms, performance estimation/testing and hyperparameter tuning. Among them, machine learning algorithms are able to characterise data for the sake of classification, regression, clustering, and outlier detection. In the present study, classification related machine learning algorithms are considered since the problem can be considered it is indispensable to classify unknown data with decent prediction.

Decision tree, ensemble decision tree (boosted and bagged), SVM (support vector machine), and k-NN (k-nearest neighbours) classification are chosen among classification algorithms according to their ability to classify imbalanced data [29]. Each algorithm has several different parameters, i.e., hyperparameters, which are to be iteratively tuned according to the Bayesian algorithm in order to optimise the results [30]. The sequence in which machine learning models are applied in this study is the same as the part indicated as “Machine learning model” in Figure 1. Each classification is described in detail, as follows.

#### 5.1.1. Decision Tree

A decision tree is normally used to compare unknown data to tree structure, which consists of roots, branches, and leaves. During the root process, unknown data are input to the decision tree algorithm, and the output of the root process is transferred to various branches. Intermediate results for determining loading types of unknown data are generated based on the characteristics of training groups, which survive criteria during the branch process. The final loading types of unknown data are unveiled in the leaf process. The decision tree has two hyperparameters, i.e., the maximum number of splits and criteria, which are summarised in Table 3 [31].

#### 5.1.2. Ensemble Method

There is a high possibility that overfitting occurs, which means that the predicted results of loading types are sufficiently good for the training group and poor prediction is generated for unreferenced elements when it involves too many branch processes. To reduce the effect of overfitting, it is recommended to apply the ensemble method, which merges outcomes from several branches into one value. In the present study, boosted decision trees and bagged decision trees are considered since they are representative machine learning algorithms with ensemble methods [9]. The boosted decision tree applies the same algorithm model, which was previously used for the decision tree, iteratively and sequentially. Iterations are conducted with an updated weighting factor until the results fulfil the criterion. It requires the maximum number of splits, the number of learners, and the learning rate as its hyperparameters. A bagged decision tree needs to apply several decision trees, which are generated by bootstrapping variations of the same decision tree algorithm model simultaneously. The best result is selected among the aggregated ones based on a majority vote. The bagged decision tree needs two hyperparameters, i.e., the maximum number of splits and the number of learners. Table 4 shows the hyperparameters for the ensemble method [32].

#### 5.1.3. SVM (Support Vector Machine)

SVM aims at forming the finest suitable decision limit or boundary, known as the hyperplane, which separates n-dimensional space into loading types, making it easy to place a different point in the appropriate area. In the SVM algorithm, extreme vector points called support vectors are chosen, which help in creating a proper hyperplane. The hyperplane of SVM is defined as the best possible decision boundary out of various possible decision boundaries that accurately classifies the classes in n-dimensional space. The features of the training group determine the dimensions of the hyperplane. A hyperplane having maximum margins, which means the distance between two data points is maximum, is preferred. Kernel function, kernel scale, box constraint level, and multiclass method are hyperparameters, which are listed in Table 5 [33].

#### 5.1.4. k-NN Classification

The k-NN classification is an instance-based learning method used to classify objects based on their closest training group in the feature space. An object is classified by a majority vote of its neighbours, i.e., the object is assigned to the class that is most common amongst its k-nearest neighbours, where k is a positive integer. In the k-NN classification, the classification of a new test feature vector is determined by the classes of its k-nearest neighbours. Here, the k-NN classification is implemented using various distance metrics to locate the nearest neighbour. Number of neighbours, distance metric, and distance weight are its hyperparameters, as shown in Table 6 [34].

### 5.2. Performance Estimation and Testing

There are four types of cross-validations, i.e., k-fold, holdout, leave-p-out, and leave-one-out, in order to estimate the performance of the machine learning algorithms [35]. In the present study, k-fold cross-validation was selected among them since it is known to have the ability to reduce biases and classify loading types with a low capacity of training data [36]. Acquired training data are randomly divided into five groups. One of the groups is arbitrarily chosen for testing, the others for training, which are put into machine learning algorithms; therefore, five trials are carried out per iteration.

The performance of the machine learning algorithms is estimated using accuracy and the ROC-AUC value [37]. Accuracy at the n-th iteration, denoted by $A_{n}$, is the proportion of correct predictions made by the model out of the total number of predictions as shown in Equation (4) [38].

(4) $$A_{n}=\frac{Number\;of\;correct\;predictions}{Total\;number\;of\;predictions}\times 100$$

Accuracy is a widely used metric because of its simplicity and effectiveness; however, it contains the mixed information of tension, compression, and shear loading types. According to the possibility of imbalanced training data, which are produced from preliminary FE crash analysis, ROC-AUC might alleviate the misleading results of the imbalanced data [39]. ROC-AUC is suitable for estimating imbalanced loading type data since it can separately show individual values for each loading type among the training group. The ROC curve comes from ratios based on correctly predicted data and incorrectly predicted data, and the AUC means the area under the ROC curve. Generally, AUC takes values from 0 to 1, where a value of 0 indicates a perfectly inaccurate estimation, and a value of 1 reflects a perfectly accurate estimation. A value of less than 0.5 suggests no discrimination, 0.7 to 0.8 is considered acceptable, 0.8 to 0.9 is considered excellent, and more than 0.9 is considered outstanding [40,41]. The convergence criterion ($C_{crit})$ is defined as the change in accuracy, as shown in Equation (5) [42].

(5) $$C_{crit}=\left|\frac{A_{n}-A_{n-1}}{A_{n}}\right|$$

In order to meet the highest performance, the value for the convergence criterion is set to be 0.1. When the results are not satisfactory, hyperparameters are tuned based on the Bayesian optimisation algorithm. Iterative performance estimation and testing are conducted simultaneously.

### 5.3. Hyperparameter Tuning with Bayesian Optimisation Algorithm

As shown in Figure 1, inside the red dotted box with the name of the machine learning model, hyperparameters are tuned through the Bayesian optimisation algorithm, which is famous for being more effective compared to grid search or random search, and iterations are conducted until the lowest error or the highest performance is obtained on the condition that the results are not satisfactory [43,44]. The main feature of the optimisation technique is to maximise the objective function, which is denoted by f along with hyperparameters as its independent variables. The output of the objective function is the performance of machine learning algorithms. Each iteration needs updating or tuning the hyperparameters within their own domain, which is represented by X. Accordingly, the Bayesian optimisation algorithm can be explained as Equation (6):

(6) $$x^{*}=\arg\underset{x\in X}{\max}f\left(x\right)$$

where x denotes a set of hyperparameter values in the domain X, and $x^{*}$ is the hyperparameter value set that maximises the performance, i.e., the output of the objective function f [45]. Hyperparameters are tuned by the Expected-Improvement-Per-Second Plus, which provides the fastest speed of the convergence criterion as well as prevents overexploiting possible ranges of hyperparameter domains from being illuded by the local maximum [46]. When the value of the convergence criterion becomes lower than 0.001, hyperparameter tuning is completed. Table 7 indicates the completely tuned hyperparameter values, which maximise the performance of each algorithm. The learning rate in the Boosted decision trees, Kernel scale, and box constraint level of SVM are displayed with a precision of four decimal points.

Table 8 and Table 9 show the highest performances, i.e., accuracy and ROC-AUC value, of 2-D and 3-D implementations. All values in Table 8 and Table 9 are the average of the top, front, chamfered, bottom, rear, and rib faces, both for accuracy and the ROC-AUC. The accuracy of the three-dimensional implementation is higher than that of the two-dimensional implementation. The k-NN classification shows the highest accuracy both for two-dimensional and three-dimensional implementations. As shown in Table 8 and Table 9, predicted ROC-AUC values for shear-dominant loading types are the smallest compared with either tensile or compressive ones, since the portion of shear-dominant reference elements is the lowest.

## 6. Prediction and Mapping of Loading Type of Unreferenced Elements

The k-NN is applied in order to predict loading types of unreferenced finite elements in the PIC bumper beam with the coordinate values of target locations since it is revealed to be the most excellent classification both for 2-D and 3-D implementations from the comparison of the resultant performance of machine learning.

**Figure 6:**
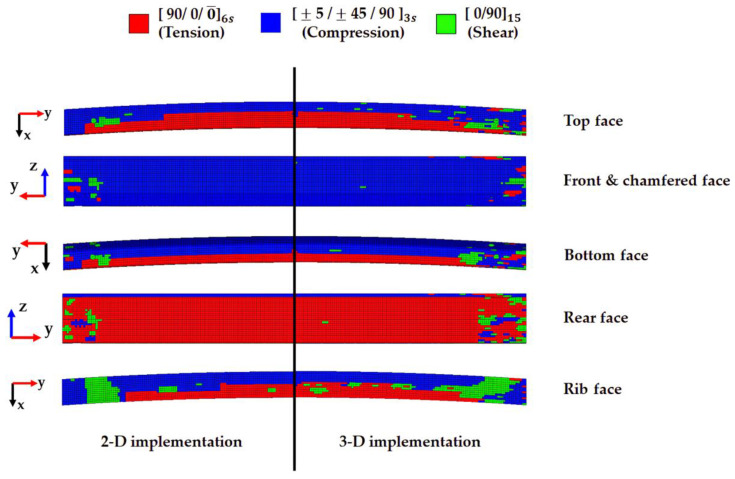
Mapping results –based on 2-D implementation vs 3-D implementation.

Predicted results contain loading types and locations for unreferenced elements; therefore, robust stacking sequences against each loading type are mapped into the FE model, as shown in Figure 6. In the meantime, robust stacking sequences against each loading type are listed in Table 10 [47].

While machine learning algorithms predict dominant loading types at a certain face, only training data are obtained from reference elements, which are located at the same face during 2-D implementation. On the other hand, training data from reference elements, which are located at various faces depending on the metric values, are used for dominant loading type prediction in 3-D implementation, as previously mentioned. As expressed in Figure 6, it is observed that three different loading types are mixed together at both ends, which are firmly connected to rectangular cross-sectional bumper crash boxes.

As a result, 42.3%, 53.2%, and 4.5% portions of tension-, compression-, and shear-dominant loading types are predicted for the entire PIC bumper beam FE model from 2-D implementation, whilst 42.1%, 50.4%, and 7.5% of tension-, compression-, and shear-dominant loading types from 3-D implementation are predicted for the same bumper beam FE model. Also, each loading type area difference is conspicuous in the rib face, i.e., tension-dominant area from 3-D is 6.5% larger than that from 2-D, compression-dominant area from 3-D is 12.5% smaller than that from 2-D, and shear-dominant area from 3-D is 5.9% larger than that from 2-D, as expressed in Figure 6. Meanwhile, loading type areas are the most similar to 2-D and 3-D on the front face. These results are summarised in Table 11.

## 7. Bending Strength Evaluation of the PIC Bumper Beam

IIHS bumper crash analyses were performed using ANSYS LS-DYNA (ANSYS, Inc.) in order to evaluate the bending strength of the PIC bumper beam based on the 2-D and 3-D implementations employing a machine learning model as well as a composite bumper beam with a conventional stacking sequence of ${\left[0/\pm 45\right]}_{5S}$ for comparison’s sake. A fully integrated shell formulation is selected to express nonlinear anisotropic behaviour without hourglass mode under the function of improved transverse shear treatment for the composite beam FE model [48]. The enhanced composite damage type material model, which is frequently used for describing material anisotropy with the help of the laminated shell theory, is selected for the whole composite part [25,49,50]. Table 12 shows the material properties and damage parameters that are used as inputs for the T700/2510 carbon fibre epoxy composite [51,52]. Element size, initial velocity, vehicle mass, and miscellaneous details are the same as those used in the preliminary IIHS bumper beam FE crash analysis.

**Figure 7:**
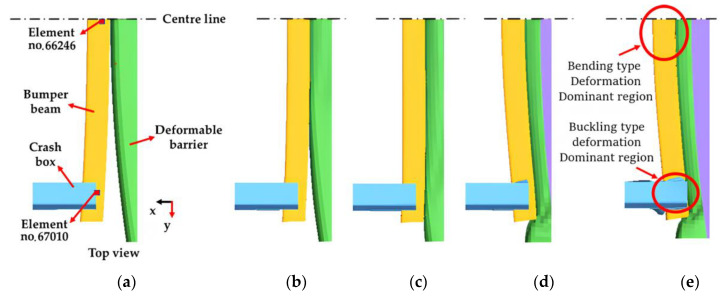
Deformation of the PIC bumper beam (3-D implementation): (**a**) Time: 0 s, C.O.G. displacement: 0 mm, (**b**) time: 0.009 s, C.O.G. displacement: 24 mm, (**c**) time: 0.02 s, C.O.G. displacement: 54 mm (**d**): time: 0.035 s, C.O.G. displacement: 80 mm, (**e**) time: 0.045 s, C.O.G. displacement: 107 mm.

The deformation of the PIC bumper beam and crash box is illustrated in Figure 7. From Figure 7b, pure bending deformation of the bumper beam is observed until 0.009 s (C.O.G. displacement: 24 mm), while buckling type crash box deformation starts at 0.02 s (C.O.G. displacement: 54 mm), as shown in Figure 7c. Deformation of the crash box initiates from the inner part and propagates to the outer part owing to the convex shape of the IIHS barrier. The bumper beam deforms to become a straight shape along with the y-axial direction, and the visible main deformation of the crash box occurs at 0.035 s (C.O.G. displacement: 80 mm), as depicted in Figure 7d. In Figure 7e, the maximum bending deformation happens both in the bumper beam and crash box at 0.045 s (C.O.G. displacement: 107 mm), and the elastic spring back starts from this moment. Meanwhile, it is observed that two representative types of deformation have occurred in the bumper beam during crash simulation, i.e., deformation due to bending at the centre part and buckling type along with cross-sectional direction at both ends, which are connected to the crash box, as depicted in Figure 7e.

Tsai–Wu indexes [53] are calculated for specific elements, which are exposed to comparatively higher loading. The no. 66,246 element and the no. 67,010 element are chosen from the centre part and the RH end part of the composite bumper beam, respectively, as visible in Figure 7a, since these parts undergo severer deformation during FE crash analyses.

**Figure 8:**
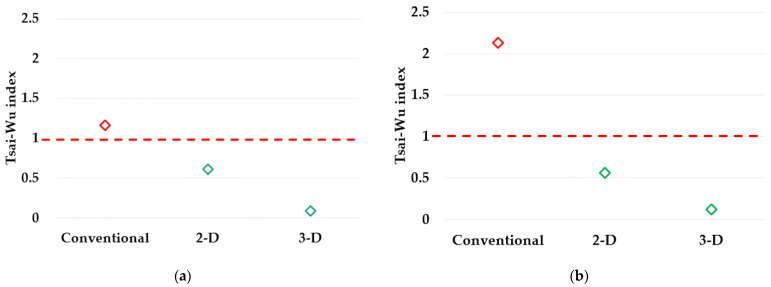
Tsai-Wu index: (**a**) Centre part (element no. 66246), (**b**) end part (element no. 67010).

The dominant loading type for the No. 66,246 element and the No. 67,010 element was found to be “shear”, by the 3-D implementation, but the 2-D implementation predicts “compression” for these elements. As a result, the Tsai–Wu indexes for the no. 66,246 element and the no. 67,010 element are shown in Figure 8.

**Figure 9:**
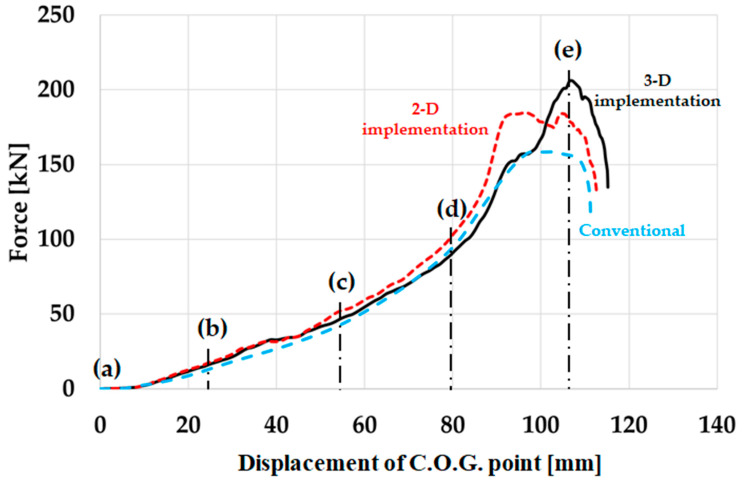
Force-displacement curve of composite bumper beams.

In Figure 8, the index, which is calculated based on the conventional composite beam, is found to be exceeding 1. The index for 2-D is close to 1, but the index for 3-D is the lowest, i.e., conventional composite beams experience fracture, while PIC bumper beams are safe under the same level of external loading. It is found that the conventional stacking sequence does not sufficiently respond to external loading, and the PIC bumper beams are safe and effective. In the meantime, the Tsai–Wu indexes reveal that the PIC beam of 3-D implementation is safer compared with that of 2-D implementation. The force–displacement of C.O.G. point curve results of conventional composite bumper beams and PIC bumper beams are plotted in Figure 9. Deformation stages (a), (b), (c), (d), and (e) in Figure 7 are marked in Figure 9.

The force curve slope increases at point (d) because crash boxes and both end parts of the composite bumper beam start to deform. A higher external load-resisting capability of a composite bumper beam is dependent on the proper stacking sequences of both end parts.

The maximum bending strength of the conventional composite bumper beam is 158 kN, while that of the PIC bumper beam based on the 2-D implementation is 184 kN, and that of the 3-D implementation is 206 kN, i.e., the bending strength of the PIC bumper beam of the 3-D implementation is about 10.4% and 23.0% higher than that of the 2-D implementation and that of the conventional stacking sequence, respectively. The PIC bumper beams with a machine learning model show superior bending strength to conventional composite bumpers. As previously observed, the PIC bumper beam of the 3-D implementation shows higher external load-resisting ability compared to that of the 2-D implementation. Therefore, it is found that the 3-D implementation is more effective in assigning proper stacking sequences to exact places of the composite bumper beam. If PIC bumper beams were designed to target the same bending strength level as the conventional composite bumper beam, 31% and 33% weight-saving effects could be achieved, as summarised in Table 13.

From a crashworthiness point of view, conventional composite bumper beams absorb 6980 J during the IIHS bumper crash analysis. Meanwhile, 2-D-implemented, and 3-D-implemented PIC bumper beams absorbed 8230 J and 8260 J, respectively. The PIC bumper beam of 3-D implementation also shows slightly higher energy absorption characteristics than either conventional or 2-D implemented composite bumper beams. Therefore, it is certain that the PIC bumper beam design with machine learning is effective in reducing the possibility of structural failure as well as increasing bending strength. The 3-D implementation produces better results compared with the 2-D implementation since it is preferable to choose the external loading type information which is achieved from surroundings when the target elements are located either at corner or junction of planes instead of using information came from the same plane of target.

## 8. Conclusions

Employing machine learning models, a PIC bumper beam is proposed in order to improve the bending strength and structural lightweight effect, which are proved by FE crash simulations regarding the bumper test protocol suggested by IIHS. The composite bumper beam undergoes two major types of deformation, i.e., bending-type deformation at the centre part and buckling-type deformation along with cross-sectional direction at both ends, which are connected to a crash box. Two- and three-dimensional implementations are provided by machine learning models, which determine the stacking sequences of each finite element in the PIC bumper beam. It was found that the PIC bumper beams have a higher bending strength; however, the conventional composite bumper beam does not sufficiently withstand external loading. In the meantime, the Tsai–Wu indexes reveal that the PIC bumper beam of the 3-D implementation is safer compared with that of the 2-D implementation. Also, the dominant loading type, which is predicted based on the 3-D implementation, for the centre part and both end parts is found to be shear, which is the correct loading type, but the 2-D implementation predicts compression for the same parts.

Bending strength of 3-D implementation is about 10.4% and 23.0% higher than that of 2-D implementation and that of the conventional stacking sequence. The PIC bumper beam of 3-D implementation shows higher external load-resisting capability compared to that of 2-D implementation. Therefore, it is found that the 3-D implementation is more effective to assign proper stacking sequences into exact places of composite bumper beam. If PIC bumper beams were designed targeting the same bending strength level of conventional composite bumper beam, 31% and 33% of weight saving effects could be achieved. It is certain that the PIC bumper beam design with machine learning is effective to reduce the possibility of structural failure as well as increasing bending strength.

From a crashworthiness point of view, the conventional composite bumper beam absorbs 6980 J during the IIHS bumper crash analysis. Meanwhile, 2-D-implemented and 3-D-implemented PIC bumper beams absorb 8230 J and 8260 J, respectively. The PIC bumper beam of 3-D implementation also shows slightly higher energy absorption characteristics than either conventional or 2-D-implemented composite bumper beams. The 3-D implementation produces better results compared with the 2-D implementation since it is preferable to choose loading-type information, which is achieved from surroundings when the target elements are located either at corners or junctions of planes, instead of using information that comes from the same plane as the target elements.

## Figures and Tables

**Table 1:** Mechanical properties of Aluminium alloy 7021.

Mechanical Properties	Value
Density ρ	2700 kg/m^3^
Young’s modulus E	70 GPa
Poisson’s ratio ν	0.3
Yield stress Y	360 MPa

**Table 2:** Boundary conditions and vehicle mass in the IIHS crash analysis.

	Boundary Condition	Mass
Bumper beam	10 km/h	2200 kg on C.O.G.
Crash box		
Vehicle		
IIHS deformable barrier	Deformable (Front) Stationary (Rear wall)	-

**Table 3:** Hyperparameters. for decision tree method.

Machine Learning Model	Hyperparameter	Variable
Decision tree	Max. number of splits	1~1209
	Split criterion	Gini’s diversity index Towing rule Maximum deviance reduction

**Table 4:** Hyperparameters for ensemble method.

Machine Learning Model	Hyperparameter	Variable
Boosted decision tree	Max. number of splits	1~1209
	Number of learners	10~500
	Learning rate	0.001~1
Bagged decision tree	Max. number of splits	1~1209
	Number of learners	10~500

**Table 5:** Hyperparameters for SVM method.

Machine Learning Model	Hyperparameter	Variable
SVM	Kernel function	Gaussian Linear Quadratic Cubic
	Kernel scale	0.001~1000
	Box constraint level	0.001~1000
	Multiclass method	One-vs-One One-vs-All

**Table 6:** Hyperparameters for k-NN classification.

Machine Learning Model	Hyperparameter	Variable
k-NN classification	Number of neighbors	1~605
	Distance metric	City block Chebyshev Correlation Cosine Euclidean Hamming
	Distance weight	Equal Inverse Squared inverse

**Table 7:** Optimised hyperparameters using Bayesian algorithms.

Machine Learning Model	Hyperparameter	Values
Decision tree	Max. number of splits	17
	Split criterion	Gini’s diversity index
Boosted decision trees	Max. number of splits	1149
	Number of learners	50
	Learning rate	0.8987
Bagged decision trees	Max. number of splits	694
	Number of learners	214
SVM	Kernel function	Gaussian
	Kernel scale	30.2498
	Box constraint level	3.8962
	Multiclass method	One-vs-One
k-NN classification	Number of neighbors	4
	Distance metric	City block
	Distance weight	Squared inverse

**Table 8:** Performances of 2-dimensional implementation.

Machine Learning Model	Accuracy (%)	ROC-AUC		
		Tension	Compression	Shear
Tree	84.2	0.85	0.88	0.73
Boosted decision trees	85.1	0.89	0.92	0.75
Bagged decision trees	81.1	0.90	0.91	0.75
SVM	85.9	0.89	0.90	0.62
k-NN classification	86.0	0.90	0.92	0.75

**Table 9:** Performances of 3-dimensional implementation.

Machine Learning Model	Accuracy (%)	ROC-AUC		
		Tension	Compression	Shear
Decision tree	85.4	0.95	0.94	0.80
Boosted decision tree	85.1	0.98	0.97	0.87
Bagged decision tree	85.3	0.98	0.97	0.87
SVM	86.2	0.96	0.94	0.62
k-NN classification	86.3	0.98	0.97	0.87

**Table 10:** Robust stacking sequences against each loading type.

Dominant Loading	Robust Stacking Sequence
Tension	${[90/0/\bar{0}]}_{6s}$
Compression	${[\pm 5\;/\pm 45/90]}_{3s}$
Shear	${\left[0/90\right]}_{15}$

**Table 11:** Predicted dominant loading type—2-D implementation vs. 3-D implementation.

Face	2-D implementation			3-D implementation		
	Tension	Comp.	Shear	Tension	Comp.	Shear
Top	43.5%	53.3%	3.2%	41.2%	50.2%	8.6%
Front & chamfered	0.8%	97.5%	1.8%	2.1%	95.9%	2.0%
Bottom	20.7%	30.4%	2.1%	15.8%	29.5%	7.9%
Rear	95.5%	2.0%	2.5%	92.0%	2.8%	5.2%
Rib	30.0%	54.4%	15.6%	36.6%	41.9%	21.5%
Total	42.3%	53.2%	4.5%	42.1%	50.4%	7.5%

**Table 12:** Material properties of T700/2510 carbon epoxy composite [51,52].

Properties	Values
Density	1520 kg/m^3^
Longitudinal modulus	126 GPa
Transverse Modulus	8.40 GPa
Shear modulus	4.23 GPa
Major Poisson’s ratio	0.31
Axial tensile strength	2172 MPa
Axial compressive strength	1450 MPa
Transverse tensile strength	49 MPa
Transverse compressive strength	199 MPa
In-plane shear strength	155 MPa
Softening reduction factor for material strength in crashfront element	0.57
Softening for fibre tensile strength	0.5
Reduction factor for compressive fibre strength after matrix failure	1.2

**Table 13:** Mass of same bending strength level composite bumper beam.

Design Method	Bumper Beam Mass (kg)	Weight Saving Effect (%)
Conventional	3.36	-
2-D implementation	2.32	31 %
3-D implementation	2.25	33 %

## Data Availability

Data are contained within the article.
